# The Evidence Effect: How Fact Boxes Shift Perceptions of Lung Cancer Screening in Austrian Medical Practice

**DOI:** 10.1002/cam4.70453

**Published:** 2024-12-09

**Authors:** Carolina Amelunxen, Michel Bielecki, Odette Wegwarth, Georg‐Christian Funk

**Affiliations:** ^1^ Karl Landsteiner Institut für Lungenforschung und Pneumologische Onkologie Vienna Austria; ^2^ Medizinische Abteilung mit Pneumologie, Klinik Ottakring and Verein zur Förderung der Wissenschaftlichen Forschung am Wilhelminenspital Vienna Austria; ^3^ Institute for Epidemiology, Biostatistics and Prevention Institute, University of Zurich Zurich Switzerland; ^4^ Heisenberg Chair for Medical Risk Literacy and Evidence‐Based Decisions, Clinic for Anesthesiology & Intensive Care Medicine Charité – Universitätsmedizin Berlin Germany; ^5^ Center for Adaptive Rationality Max Planck Institute for Human Development Berlin Germany

**Keywords:** fact box, icon array, physician education, screening

## Abstract

**Background:**

Recent results from the Dutch NELSON study have rekindled debates about the benefit‐to‐harm ratio of lung cancer screening and the comprehension of this by physicians.

**Methods:**

This research surveyed the perception and understanding of 136 Austrian physicians regarding the advantages and risks of lung cancer screening, examining the impact of educational data visualization tools, including fact box and icon array. Physicians participated in an online survey about their understanding before and after exposure to either a fact box alone or combined with an icon array.

**Results:**

The findings indicated that the fact box significantly enhanced physicians' grasp of the screening's benefits and harms, making them up to 13 times more likely to adjust their estimates within a predefined range. Notably, the intervention was more effective among physicians who initially did not recommend CT screening. However, the addition of the icon array did not offer significant improvement. Postintervention, physicians showcased better comprehension and an improved ability to offer patient‐centered advice, which may bolster adherence to lung cancer screening protocols.

**Outlook:**

Despite its insights, the study's cross‐sectional nature and the unique cultural context underline the need for more research. Further exploration should focus on different settings and assess the real‐world implications on clinical practice and patient outcomes.

## Graphical abstract

This manuscript investigates the impact of educational visualization tools on physicians' understanding of lung cancer screening. The study surveyed 136 Austrian physicians and found that exposure to fact boxes significantly improved their comprehension of the benefits and harms of low‐dose CT screening, making them more likely to adjust their estimates accurately. However, adding an icon array did not further enhance understanding, and the study suggests the need for further research to explore the long‐term effects and applicability of these tools in different settings.

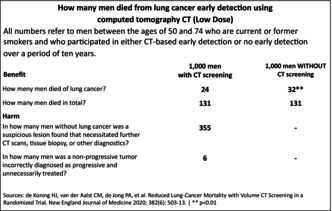

## 1 Introduction

Lung cancer is both the most common cancer globally and has the highest mortality rate among malignancies [1].

Currently, no screening method has conclusively demonstrated a reduction in overall mortality. For example, annual lung X‐rays for high‐risk individuals (smokers and ex‐smokers) have been shown to be ineffective in reducing lung cancer mortality, leading to their exclusion from guideline recommendations (PLCO study) [2].

The Dutch–Belgian NELSON trial evaluated low‐dose chest CT as a screening tool for high‐risk populations, primarily smokers and ex‐smokers. Over a 10‐year follow‐up, lung cancer mortality was 2.50 deaths per 1000 person‐years in the screening group, compared with 3.30 in the control group. Although the absolute risk reduction appears small, in the context of cancer screening, even modest reductions can be meaningful, and highlight the potential of CT screenings.

However, while lung cancer screening reduces cancer‐specific mortality, it has no effect on overall mortality, as confirmed by three meta‐analyses [3, 4, 5].

The National Lung Screening Trial (NLST) found that low‐dose computed tomography resulted in a reduction in lung cancer mortality in comparison with chest X‐rays; however, it did not find a significant decrease in overall mortality. This indicates that while LDCT may help detect and treat lung cancer earlier, it does not necessarily improve survival from all causes.

Despite evidence supporting low‐dose thoracic CT screenings, it has not been widely adopted for high‐risk patients [6]. This hesitancy is attributed to the minimal absolute mortality reduction and the high prevalence of false‐positive results, which can lead to unnecessary diagnostic interventions, overtreatment, unwarranted patient anxiety, and further costly diagnostic work‐ups, and concerns over radiation exposure [7, 8, 9, 10].

The German Society of Pneumology mandates healthcare providers to communicate all benefits and risks of lung cancer screening, including the modest survival benefits and potential risks associated with chest CT scans, to ensure informed patient decision [11, 12, 13, 14]. Given the nuanced balance of risks and benefits, shared decision‐making between healthcare providers and patients is essential. In lung cancer screening, this process is particularly important, as the decision to screen must consider individual patient risk factors and preferences.

Existing research highlights concerns about physicians' understanding of cancer screening, raising doubts about their ability to guide patients in making informed decisions. In lung cancer screening specifically, studies show significant gaps in statistical competence, with some physicians overestimating the mortality reduction from low‐dose CT by as much as sixfold [15].

So, how can this situation be improved? In the realm of risk communication, several tools have been developed that have shown efficacy in correcting patients' and physicians' misconceptions about various medical interventions [11, 16, 17].

The aim of our study was twofold. First, we assess physicians' attitudes toward low‐dose chest CT for lung cancer screening and their understanding of its benefits and harms. Second, we evaluate the effectiveness of educational tools, specifically fact boxes and icon arrays, in improving physicians' comprehension and influencing their screening recommendations. We hypothesize that these tools will correct misconceptions and promote accurate risk–benefit assessments, facilitating informed decision‐making.

## 2 Methods

In order to investigate this subject, we conducted a prospective, randomized online survey. The survey was administered using the internet‐based survey tool, “SurveyMonkey.” Physicians in this study were fully qualified members from two professional societies: the Austrian Society of Pneumology (ÖGP) and the Austrian Radiological Society (ÖRG). This ensured that all respondents had completed training either in pneumology or radiology, thereby providing responses grounded in their professional expertise.

We only included pneumologists and radiologists, as these two specialties are most familiar with lung cancer screening in Austria. Furthermore, the professional associations (ÖGP and ÖRG) recommend that screening should ideally be carried out by specialized centers consisting of pulmonologists and radiologists. For a better assessment of the advantages and disadvantages of lung cancer screening, the opinion of these two specialist groups is therefore the most relevant.

**FIGURE 1:**
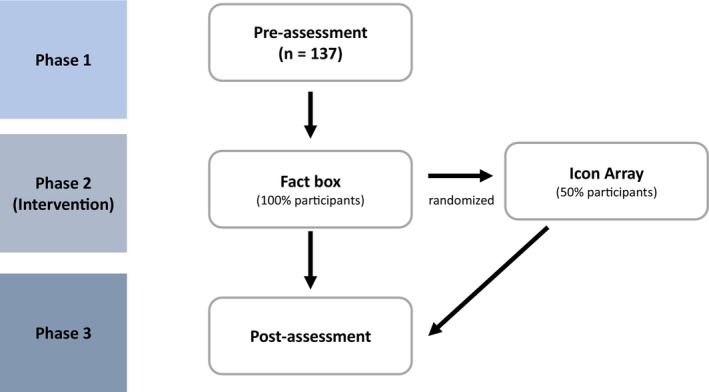
Flowchart of the study process, outlining three distinct phases. Phase 1 involves preassessment, while Phase 2 introduces the fact box intervention and an additional icon array intervention to a randomly selected half of the physicians. Phase 3 encapsulates the postintervention reassessment.

The study comprised three phases (Figure 1). Within the first phase, physicians had to answer questions about their current recommendation practices and their beliefs about the benefits and harms of chest CT for lung cancer screening.

**FIGURE 2:**
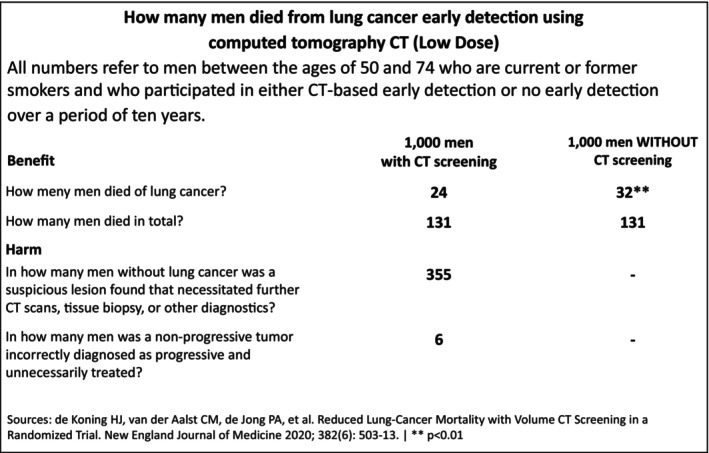
Fact box translated from the German version.

In Phase 2, all surveyed physicians were presented with a fact box that contained quantitative information about the benefits and harms of chest CT as a screening method (Figure 2). Fact boxes are tabular representations of benefits and harms framed in absolute risk terms for both screened and unscreened groups. They adhere to three key principles: completeness, balance, and transparency. They are designed to provide unbiased information, clearly depicting both benefits and risks, and using absolute rather than relative terms [18]. Fact boxes allow patients and clinicians to make informed decisions based on comprehensive and transparent data [18, 19, 20, 21]. For these reasons, we believe that the fact box is the ideal tool for communicating risk assessment and should be used as the tool of first choice.

**FIGURE 3:**
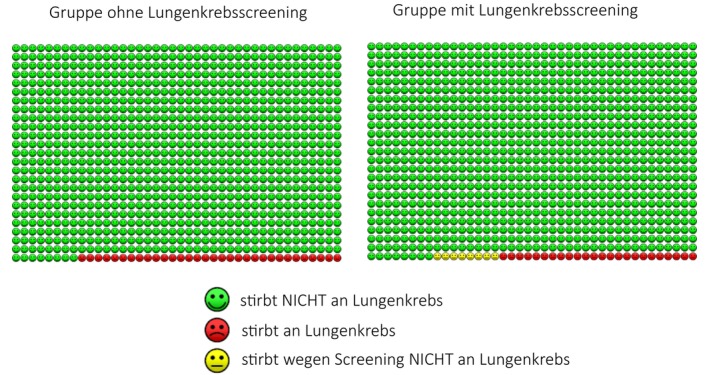
Additional icon array intervention used in randomly selected half of the physicians in Phase 2 of the study.

In addition to the fact box, a random subset of these physicians was selected via a 1:1 computerized randomization process to also receive an icon array (Figure 3). Icon arrays use visual components, such as symbols, to intuitively represent statistical values in absolute risk terms. They have been shown to help clarify complex data by visually comparing outcomes for different patient groups [22]. Whether the use of an icon array provides an additional benefit is another question posed by this study.

Immediately after exposure to the interventional material, the third phase commenced and physicians were presented with the same questions they had to answer in the initial phase in order to investigate the effects of the intervention on their benefit‐to‐harm estimations of lung cancer screening.

Our study design adopted a modified version of the questionnaire utilized by Wegwarth et al. [11] specific to lung cancer screening. The questionnaire was bifurcated into two parts: the first part solicited physicians' screening recommendation practices and their reasoning. The second part comprised seven questions assessing knowledge of current lung cancer screening data derived from the NELSON study, including mortality rates and potential harms associated with lung cancer screening.

A fact box, modeled after the one used in Wegwarth's ovarian cancer trial and using data from the NELSON study, was integrated to portray lung cancer mortality, overall mortality, and possible screening harms.

The study aimed primarily to determine whether this fact box could improve physicians' accuracy in estimating lung cancer screening outcomes, aligning their perceptions closer to NELSON study data. We also evaluated if supplementing the fact box with an icon array would further augment the physicians' risk competence.

### 2.1 Statistical Analysis

Data are represented as frequencies and percentages for categorical variables, and medians along with interquartile ranges (1st to 3rd quartiles) for continuous variables. In accordance with previously established literature, evidence‐based boundaries were set for various measures such as rates of positive test results, lung cancer mortality with and without screening, false‐positive rates, and rates of overdiagnosis.

To determine the impact of exposure to a fact box on physicians' estimates, McNemar's test was utilized using STATA (Version 17). This test allowed us to contrast the proportions of physicians whose estimates fell within these evidence‐based ranges, both before and after the exposure. We provided effect sizes, confidence intervals, and odds ratios for a comprehensive understanding of the results.

Further in our study, we aimed to discern the differential impacts of singular versus dual intervention strategies on physicians' estimations. We employed a two‐sample *t*‐test (assuming equal variances) across key variables such as doctors' estimates of mortality over the next decade with and without screening, both pre‐ and postinterventions, whether singular (fact box) or dual (fact box + icon array). The *t*‐test, grounded on the null hypothesis of identical means between groups, enabled us to scrutinize the variations in physicians' understanding of the mortality implications of chest CT screening after interventions, thereby assessing the efficacy of our intervention tools.

In addition to the *t*‐test, we employed a two‐sample Wilcoxon rank‐sum (Mann–Whitney) test to compare the distribution of values in the two intervention groups. This nonparametric test allows us to assess whether the two independent samples were selected from populations with the same distribution, thereby providing additional robustness to our analyses.

Regarding missing data, we adopted a complete‐case analysis approach. This involved disregarding any records with missing data, under the assumption that these were completely random and thus did not introduce bias into the results. We acknowledge, however, that this approach might potentially lead to a loss of information and statistical power.

To assess the robustness of our findings, we conducted a sensitivity analysis by varying the tolerance thresholds used to define accurate estimates. Initially, a threshold of ±15% around the true values was used. In the sensitivity analysis, we employed a stricter criterion by defining the range based on the square root of the true value. The sensitivity analysis was further extended by simulating scenarios where concordant and discordant pairs were underestimated and overestimated, respectively. We then recomputed the McNemar's test under these alternate conditions to determine whether the conclusions would shift. This robustness checks confirmed that the results remained consistent, reinforcing the reliability of our findings.

The study underwent review by the Institutional Review Board (IRB) commission of the Gesundheitsdienst der Stadt Wien, which determined that it did not require a full ethical review based on the provided materials. All experiments were conducted in strict accordance with relevant guidelines and regulations. The study was reviewed in accordance with the CONSORT checklist for randomized trials, and no significant deviations from the standard protocol were identified.

Additionally, we examined whether the intervention's impact differed based on physicians' initial recommendation of chest CT screening. Physicians were stratified into two groups: those who recommended CT screening and those who did not. Within each group, we compared pre‐ and postintervention estimates using the Wilcoxon signed‐rank test for paired data, as this nonparametric test is suitable for assessing changes in related samples without assuming normality. To compare the magnitude of changes between the two groups, we conducted regression analyses on the differences in estimates (postintervention minus preintervention), using the initial recommendation status as the independent variable. This approach allowed us to assess whether the intervention's effectiveness varied according to physicians' baseline screening recommendations.

### 2.2 Sample Size

We opted not to use a formal sample size calculation for practical constraints: Often in real‐world settings, especially in studies involving medical professionals, strict sample size calculations may be difficult to adhere to due to time constraints, availability of participants, or willingness to participate. In this case, focusing on maximizing participation within the available time was more feasible than adhering to a predetermined sample size.

By focusing on reaching as many doctors as possible, we maximized engagement from the target audience, which is crucial when introducing novel concepts like fact boxes in medical decision‐making. A wider reach may provide richer data for qualitative analysis and inform future more rigorously designed studies.

This approach allowed for flexibility and ensured that the study could proceed without limiting the number of valuable insights collected, while still providing meaningful, real‐world data that reflect the views of a larger portion of the medical community in Austria.

## 3 Results

### 3.1 Physician Characteristics

A total of 350 members of the Austrian Society of Pneumology (ÖGP) and 623 members of the Austrian Roentgen Society (ÖRG) were invited to participate in the survey via email. Among the contacted members, 83 ÖGP and 53 ÖRP members participated. In total, 136 physicians were included in the study. Of these, 63 received only the fact box, and after randomization, 53% also received an icon array in addition to the fact box.

### 3.2 Perceptions Toward Low‐Dose Chest CT as a Lung Cancer Screening Modality Among Physicians

Among the total of 136 physicians included in the study, approximately one‐third of the respondents (33%) reported recommending chest computed tomography (CT) as a lung cancer screening method for smokers and ex‐smokers. No significant difference w was found in recommendation behavior between the members two societies, with 35% of ÖRG members and 30% of ÖGP members endorsing CT screening (*p* = 0.651) (Table 1).

**TABLE 1:** Physician recommendations for CT screening (*n* = 136) and their reasons.

Recommends CT screen	Total *n* = 136	ÖGP *n* = 83	ÖRG *n* = 53	*p*
Yes	45 (33%)	29 (35%)	16 (29%)	0.651
No	91 (66.9%)	55 (65%)	36 (70%)	
Reasons for recommendation				
Mortality reduction	79 (58%)	47 (57%)	32 (60%)	0.72
Patients' expectation	36 (26.5%)	20 (24%)	16 (30%)	
Lung cancer incidence reduction	15 (11%)	11 (13%)	4 (8%)	0.40
Concern about legal consequences	9 (6.6%)	7 (8%)	2 (4%)	0.5
Financial incentives	2 (1.47%)	0 (0%)	2 (4%)	0.15
Guidelines	72 (52.9%)	43 (32%)	28 (20.5%)	0.85
Concern about negative consequences	46 (33.8%)	30 (36%)	16 (30%)	0.6

Physicians who endorsed CT screening cited several reasons, including the potential for mortality reduction (58%), alignment with patients' expectations (26.47%), a reduction in lung cancer incidence (11%), the concern about legal consequences (6.6%), and the influence of financial incentives (1.47%). Adherence to guidelines (52.9%) and the concern about potential negative consequences (33.8%) were also significant factors in recommending CT screening. No significant difference was observed between the responses of ÖGP and ÖRG members on these factors.

The similarities in recommendation behavior between ÖGP and ÖRG members suggest that both groups share comparable views on CT screening, though the recommendation rates remain modest in both.

### 3.3 Survey of Physicians on the Benefits and Harms of Chest CT as a Screening Method

When assessing the perceived benefits and harms of chest CT as a screening tool, physicians recognized several potential harms. The most frequently mentioned harm was iatrogenic harm, cited by 57 physicians (42%). This was followed by false‐positive results mentioned by 46 physicians (34%). Overdiagnosis and overtreatment were noted by 21 physicians (16%), false‐negative results by 7 physicians (5%), and costs by only 4 physicians (3%).

### 3.4 Estimate Summary

#### 3.4.1 Estimates of Mortality Over the Next Decade Without Screening

The intervention significantly influenced the mortality estimates, with the true value being 32 deaths per 1000 unscreened individuals. Preintervention data showed a mean of 47, SD = 73.7, and a median of 20. After the intervention, these parameters shifted closer to the true value, the mean dropping to 41.1, SD decreasing to 30.6, and the median rising to 32. Analysis showed revealed that a significant proportion (*n* = 39) of respondents adjusted their predictions to fall within the prescribed range, confirming the intervention's effectiveness (odds ratio = 13, *p* < 0.001). This demonstrates the intervention's ability to both limit overestimations and improve the accuracy of estimates (Table 2).

**TABLE 2:** Summary of physicians' estimates before and after the intervention.

Estimate	True value (per 1000)	Mean before (SD)	Median before	Mean after (SD)	Median after	Odds ratio	*p*
Mortality over the next decade without screening	32	47.00 (73.70)	20	41.10 (30.60)	32	13	< 0.001
Mortality over the next decade with screening	24	22.76 (30.97)	10	30.82 (29.07)	24	13	< 0.001
Positive chest CT screening results	385	174.89 (191.03)	100	288.09 (149.20)	350	9.54	< 0.001
Additional procedures required due to screening	355	120.45 (164.18)	50	218.86 (158.53)	275	11.3	< 0.001
Unnecessary treatment	6	24.54 (84.40)	6.5	9.52 (10.86)	6	1.83	0.2253

### 3.5 Estimates of Mortality Over the Next Decade With Screening

The true mortality rate with screening is estimated at 24 deaths per 1000 screened individuals over a decade. Preintervention estimates showed a mean of 22.76 (SD = 30.97) and a median of 10. Postintervention, the estimates shifted with the mean value increasing to 30.82 (SD = 29.07) and a median reaching 24, aligning closely with the true value of 24. Following the intervention, 39 doctors significantly adjusted their estimates to fall within the correct range (*p* < 0.001, OR = 13). The intervention led to a significant shift in central tendency and reduced variability, bringing the estimates closer to the true value and confirming the intervention's effectiveness.

### 3.6 Estimates of Positive Chest CT Screening Result

In terms of physicians' estimates for positive results from chest CT screenings, the fact box intervention had a significant impact. The true‐positive chest CT screening result is 385 per 1000 screened. Before the intervention, estimates had a mean value of 174.89 (SD = 191.03) and a median of 100, a considerable underestimation of the true value of 385.

After the intervention, the mean increased to 288.09 (SD = 149.20) and the median to 350, bringing the estimates closer to the true value. Postintervention, 41 out of 74 doctors revised their estimates within the correct range, The McNemar test indicated a significant increase in the number of physicians providing accurate estimates with an exact odds ratio of 9.54, meaning participants were 9.54 more likely to provide accurate estimates following the intervention (*p* < 0.001). The intervention produced a marked shift in the central tendency and reduced the variability of the estimates, bringing them closer to the true value, significantly increasing the number of doctors adjusting their estimates within the correct range.

### 3.7 Estimates of Additional Procedures Required due to Screening

In addressing the estimation of men without lung cancer necessitating additional procedures such as CT or biopsy following screening, the fact box intervention led to a significant recalibration in physicians' estimates. The true rate of additional procedures due to screening is 355 additional procedures per 1000 screened individuals. Before the intervention, estimates showed an overestimation with a mean of 120.45 (SD = 164.18), far from the true value of 355.

After the intervention, the mean increased to 218.86 (SD = 158.53) and the median rose to 275, moving closer to the correct value. A McNemar test confirmed the statistical significance of these shifts (*p* < 0.001). The odds ratio of 11.3 indicates that postintervention, physicians were over 11 times more likely to adjust their estimates to within the correct range. These findings highlight the fact box intervention's effectiveness in improving physicians' understanding of the frequency of additional procedures required for men without lung cancer after screening, emphasizing its educational value.

### 3.8 Estimates of Unnecessary Treatment

In examining estimates of unnecessary treatment, the fact box intervention had a smaller effect. The true rate is 6 unnecessary treatments per 1000 screened individuals. Before the intervention, the mean estimate was 24.54 (SD = 84.40), well above the true value of 6. After the intervention, the mean dropped to 9.52 (SD = 10.86), closer to the correct value but without statistical significance (*p* = 0.2253). Only six physicians adjusted their estimates to fall within the correct range postintervention, with an odds ratio of 1.83, suggesting a slight increase in the likelihood of aligning estimates, though this change was not statistically significant.

### 3.9 One Intervention (Fact Box) Versus Two Interventions (Fact Box + Icon Array)

The results from the two‐sample *t*‐test showed no significant difference in the mean estimates between the group that received one intervention (M = 42.06, SD = 29.92, *n* = 36) and the group that received two interventions (M = 41.15, SD = 31.16, *n* = 50). The mean difference was 0.91 with a standard error of 6.70, and the 95% confidence interval of the difference ranged from −12.41 to 14.23. With a *p* value of 0.8923 the difference in means was not statistically significant. Thus, the null hypothesis that the two groups' mean estimates are equal could not be rejected.

The results from the Wilcoxon rank‐sum test also found no significant difference in the distributions between the two intervention groups (*z* = 0.265, *p* = 0.7912). This suggests that the impact of a single intervention versus two interventions on the physicians' estimates did not differ significantly.

### 3.10 Sensitivity Analysis

The sensitivity analysis, which applied a tolerance range of ±15% around true values, confirmed the robustness of our findings. When adjusting for potential over‐ and underestimation of concordant and discordant pairs, the *p* value shifted from 0.0017 to 0.0032 in the exaggerated scenarios. This shift confirmed that the intervention's effect on improving physicians' estimates remained statistically significant.

**FIGURE 4:**
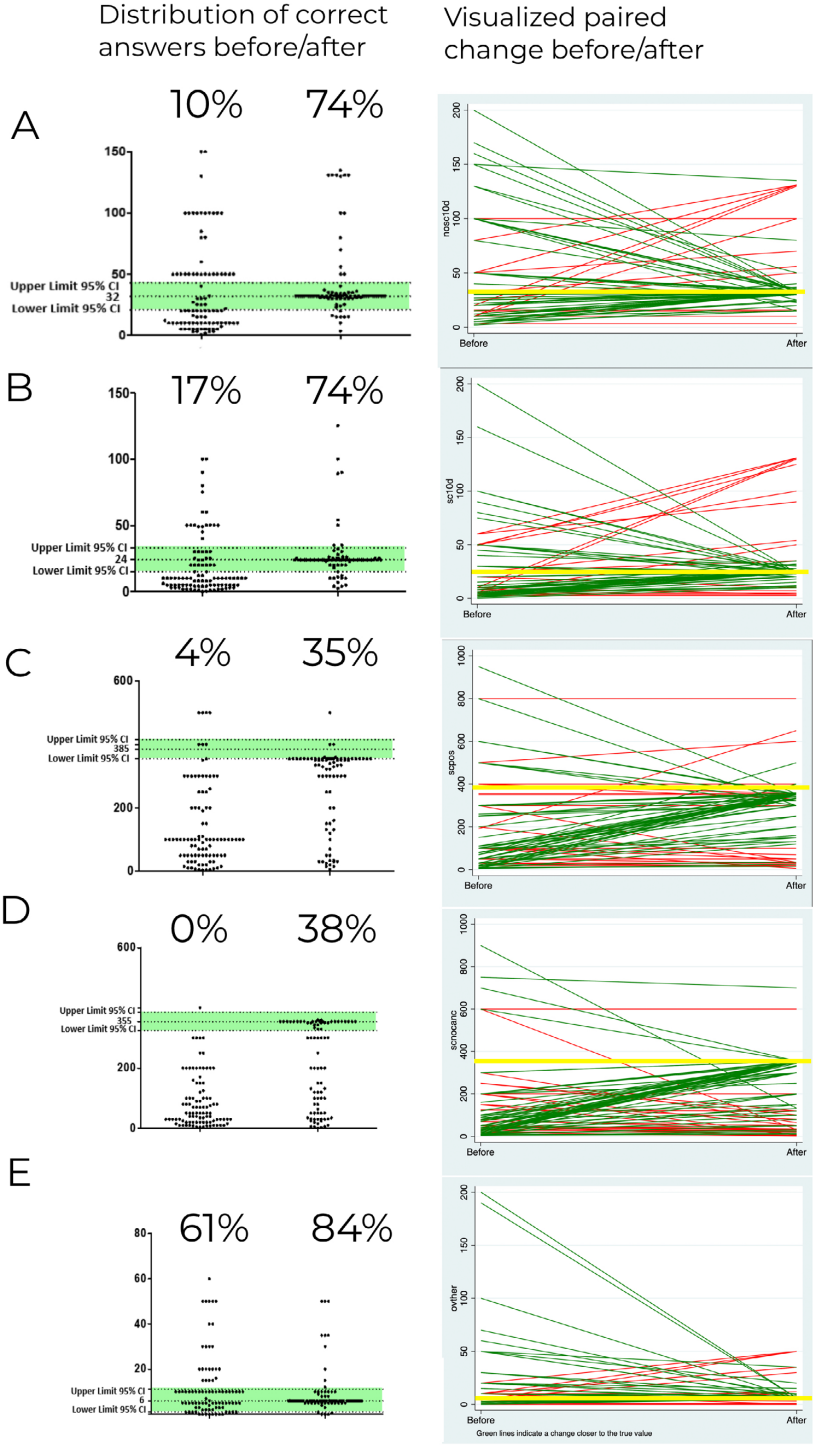
Impact of a fact box intervention on the accuracy of doctors' predictions. (A) Estimates of positive chest CT screening result. (B) Estimates of mortality over the next decade with screening. (C) Estimates of positive chest CT screening result. (D) Estimates of additional procedures required due to screening. (E) Estimates of unnecessary treatment.

Figure 4 delineates the impact of a fact box intervention on the accuracy of doctors' predictions. To the left, we present a scatter plot distribution illustrating the absolute correct answers both prior to and subsequent to the intervention. This offers a clear picture of the distribution and shift in the correctness of responses. The right portion of the figure manifests a paired plot, offering a comprehensive portrayal of the alteration in responses. Each line corresponds to an individual physician, with the color of the line signifying the effect of the intervention on the proximity of the physician's answer to the true value. Green lines correspond to individuals whose postintervention answers were closer to the correct value than their preintervention counterparts. Conversely, red lines represent instances where the intervention did not improve, and, in fact, distanced the physician's response from its true value (yellow line).

### 3.11 Changes in Clinicians' Estimates Before and After the Intervention by Initial CT Screening Recommendation

Among the 55 physicians who did not recommend CT screening, the intervention significantly improved estimates for mortality with screening (true value = 24 per 1000 screened; *p* = 0.0023), positive CT results (true value = 385 per 1000 screened; *p* < 0.0001), and additional procedures required due to screening (true value = 355 per 1000 screened; *p* < 0.0001). In contrast, for the 28 physicians who initially recommended CT screening, the intervention did not produce significant changes in estimates for these variables (*p* > 0.05). Regression analyses further indicated that the improvement in estimates for additional procedures required was significantly greater among physicians who did not recommend CT screening (coefficient = −97.55, *p* = 0.0472). These results suggest that the intervention was more effective in improving risk perception among physicians who were initially less inclined to recommend CT screening (Table 3).

**TABLE 3:** Changes in clinicians' estimates before and after the intervention by initial CT screening recommendation.

Estimate	Group	Mean before	Median before	Mean after	Median after	Paired test *p*‐value
Mortality over the next decade without screening	Did not recommend (*n* = 55)	46.91	20	41.02	32	0.2116
	Recommended (*n* = 28)	76.51	23.5	40.01	32	0.5159
Mortality over the next decade with screening	Did not recommend (*n* = 55)	22.02	10	29.89	24	0.0023
	Recommended (*n* = 28)	37.66	10	32.7	24	0.124
Positive chest CT screening results	Did not recommend (*n* = 54)	161.44	100	289.87	344	< 0.0001
	Recommended (*n* = 27)	213.11	100	262.57	328.5	0.2186
Additional procedures required due to screening	Did not recommend (*n* = 54)	99.13	50	219.09	300	< 0.0001
	Recommended (*n* = 27)	173.93	40	206.7	150	0.3471
Unnecessary treatment	Did not recommend (*n* = 53)	23.89	6	9.97	6	0.4564
	Recommended (*n* = 28)	22.21	5	7.75	6	0.6442

## 4 Discussion

While our study is the first to investigate fact box and icon array interventions in the context of lung cancer screening, prior research has demonstrated the broader effectiveness of fact boxes in enhancing medical decision‐making. Wegwarth et al. showed significant improvements in gynecologists' understanding of ovarian cancer screening after a fact box intervention, underscoring their potential for strengthening evidence‐based practice. Similar tools, such as case‐based modules for early‐stage arthritis screening [23], led to considerable learning, although retention declined over time. Other studies have highlighted the need for continuous training to maintain knowledge, particularly in critical areas like emergency and cardiovascular care [24]. Focus groups have also validated fact boxes as valuable aids for communicating the benefits and risks of medical treatments [25]. Collectively, these findings support the role of fact boxes as effective tools for improving physicians' comprehension and retention of critical medical information.

Interestingly, the intervention's impact varied depending on whether physicians initially recommended CT screening. Physicians who were initially hesitant about recommending CT screening showed significant improvements in their estimates for mortality with screening, positive CT results, and additional procedures (*p* < 0.01 for all). Conversely, those who initially supported CT screening did not show significant changes (*p* > 0.05). This finding highlights the potential of targeted interventions like fact boxes to shift perspectives, particularly among those less inclined toward screening, and suggests that future educational efforts should prioritize such groups to maximize their impact.

The recent publication of the European NELSON study, revealing a reduction in mortality due to lung cancer screening akin to the results from the US NLST study, has renewed discussions across Europe regarding the implementation of standardized screening programs. The prevalent demand for an effective early‐stage diagnostic method for lung cancer is clear from the responses of our study participants, with a third of the respondents endorsing the use of thoracic computed tomography, a technique not yet widely recognized in current guidelines.

The hesitance toward the adoption of national screening programs arises from a myriad of factors. Prominent among these are concerns about potential harms, such as radiation exposure and false positives. Intriguingly, before any intervention, most physicians' estimates of the benefits and risks of lung cancer screening were not consistent with evidence‐based data.

Our intervention using the fact box significantly improved these estimates, including perceptions of the impact on mortality. This finding underlines the value of enhancing physicians' statistical risk competence to provide comprehensive patient information and facilitate informed decision‐making. With the improved understanding, physicians are likely to engage more effectively in shared decision‐making processes and offer more patient‐centered counseling. This change could potentially enhance the acceptance and adherence to lung cancer screening among at‐risk patients.

Upon reviewing the data from the NELSON study presented in the fact box, we observed a considerable improvement in respondents' comprehension of the screening data. This comprehension pertained to the understanding of mortality rates in the screening versus the nonscreening groups, false‐positive findings, as well as the risks of overdiagnosis or overtreatment. Therefore, the fact box proved to be an effective tool in facilitating comprehension of statistical data.

In contrast, the additional use of the icon array did not seem to offer further value in this context, prompting us to propose its omission from doctor‐facing communications regarding screening data. It should be noted, however, that our study may have been underpowered due to the number of participants. This limits the conclusiveness of the findings regarding the efficacy of the icon array. Furthermore, our study did not directly compare the individual effectiveness of the fact box and the icon array. Thus, it remains an open question whether the icon array alone could be equally efficient in conveying information. Future studies with larger sample sizes and direct comparisons between different information visualization tools are required to answer this question.

Additionally, our findings should encourage the medical community to reevaluate how data is communicated to healthcare providers. As our results suggest, the current methods may not be as effective as we assume. Integrating tools like the fact box into medical education and continuing professional development programs could.

Our study has several limitations that warrant consideration. First, the observed improvements in understanding, while significant, are based on physicians' self‐reported estimates. The translation of this enhanced understanding into clinical practice and its impact on patient outcomes were not within the scope of this study and need to be assessed in future research.

Additionally, the cross‐sectional nature of our study limited our ability to evaluate the long‐term retention of these changes. Future longitudinal studies are required to assess how sustained the improvements from the fact box intervention are over time.

Our study focused specifically on lung cancer screening, and while the fact box intervention showed value in this context, further studies in different clinical areas and health interventions will be essential to determine whether these findings can be generalized. Broad conclusions from a single study should be made cautiously, and further research is needed to refine strategies that improve physicians' understanding of medical statistics.

Lastly, the generalizability of our results may be limited, as the study only involved Austrian physicians. Replicating the study in other cultural and healthcare settings with a more diverse sample of physicians would help extend the relevance of our findings and provide a broader understanding of the fact box's efficacy across different healthcare systems.

Our study provides compelling evidence that fact boxes can improve physicians' understanding of key data on lung cancer screening, enabling more informed decision‐making. These tools have particular promise for improving estimates among clinicians who are initially less likely to recommend screening, thus addressing gaps in risk perception. However, further research is needed to assess whether these improved understandings influence clinical practices and patient outcomes. Broader studies that replicate these findings in different healthcare contexts and with other medical interventions will help refine the use of fact boxes as a core tool for medical education and patient care.

## Author Contributions

**Carolina Amelunxen:** conceptualization (equal), methodology (equal), writing – original draft (equal). **Michel Bielecki:** formal analysis (equal), software (equal), writing – original draft (equal), writing – review and editing (equal). **Odette Wegwarth:** conceptualization (equal), supervision (equal), validation (equal). **Georg‐Christian Funk:** conceptualization (equal), data curation (equal), formal analysis (equal), investigation (equal), methodology (equal), project administration (equal), writing – review and editing (equal).

## Conflicts of Interest

The authors declare no conflicts of interest.

## Supporting information


Supporting Information S1.

## Data Availability

The data and do files supporting the findings of this study are available in the provided GitHub repository. To interpret, replicate, and build upon the results reported in this article, interested researchers can access the minimal necessary dataset at the following link: [https://github.com/kushiel42/Evidence_Paper]. We ensure that all data shared respects individual privacy and meets ethical standards.
