# Supplementary material for: The Evidence Effect: How Fact Boxes Shift Perceptions of Lung Cancer Screening in Austrian Medical Practice
Source: Cancer Med. 2024 Dec 9;13(23):e70453. doi: 10.1002/cam4.70453 (PMC11626479; doi:10.1002/cam4.70453)
Supplement: Supplementary file 1 — Supporting Information S1. [file CAM4-13-e70453-s001.docx]

**ORIGINAL SURVEY INSTRUMENT (ENGLISH TRANSLATION BELOW)**

„Sind die Einschätzungen Österreichischer Lungenfachärzte zum Lungenkrebsscreening durch eine Fact-Box beeinflussbar?“

**Fragebogen**

Sehr geehrte Frau Kollegin/sehr geehrter Herr Kollege,

Eine Vielzahl an medizinischen Entscheidungen werden nicht in Situationen völliger Sicherheit getroffen, sondern fallen in die zahlreichen Graubereiche der Wahrscheinlichkeiten. Im Rahmen dieser Studie möchten wir einen dieser Bereiche untersuchen und von Ihnen als Österreichische Expertinnen und Experten wissen, wie Sie das Lungenkrebsscreening bewerten. Auf Basis dieser Befragung wollen wir herausfinden, in welcher Weise die Vor- und Nachteile von Lungenkrebsscreening am besten für alle Beteiligten aufbereitet und dargestellt werden sollen.

Zur Beantwortung dieser Forschungsfrage bitten wir Sie, sich kurz Zeit zu nehmen und Fragen zu Ihrer Einstellung und Empfehlungspraxis zu Thorax-CTs als Screening Methode zu beantworten. Im Anschluss erhalten Sie eine Fact-Box mit numerischen Informationen zu Nutzen und Schaden dieser Methode. Die Hälfte der Teilnehmerinnen und Teilnehmer erhält außerdem dieselbe Information zusätzlich zur Fact-Box graphisch in Form eines sogenannten Cates plot aufgearbeitet. Nach Durchsicht dieser Informationen bitten wir Sie den Fragebogen erneut zu beantworten. Ihre Teilnahme an diesem Forschungsprojekt ist freiwillig. Sie können jederzeit, ohne Angabe von Gründen, die Befragung abbrechen.

Alle Studienteilnehmerinnen und -teilnehmer werden mit einer fortlaufenden Nummer codiert (pseudonymisiert). Die auszuwertenden Daten werden nur mit diesem Code versehen in einer Excel-Tabelle auf einem Laufwerk des Karl Landsteiner Instituts für Lungenforschung und Pneumologische Onkologie, Klinik Ottakring mit Zugriffsbeschränkung gespeichert und anschließend ausgewertet. Nur autorisierte Personen haben Zugriff auf die Originaldaten. Nach Abschluss der Studie wird die Datei gelöscht.

Vielen herzlichen Dank für Ihre Teilnahme an dieser Befragung!

**Empfehlungsverhalten/ Gründe für Empfehlung**

**Frage 1**

Empfehlen Sie routinemäßig eine Thorax-Computertomographie als Lungenkrebs Screening bei Rauchern und Ex-Rauchern?

- Ja
- Nein

**Frage 2**

Welche der folgenden Gründe beeinflussen Ihre Entscheidung zur Empfehlung/ Nicht-Empfehlung einer Thorax-Computertomographie als Screening Methode für Lungenkrebs?

|  |  | **Dies beeinflusst meine Entscheidung** | **Dies beeinflusst meine Entscheidung NICHT** |
| --- | --- | --- | --- |
| **1.** | Reduktion der Lungenkrebs Mortalität durch das Screening. |  |  |
| **2** | Reduktion der Lungenkrebs Inzidenz durch das Screening. |  |  |
| **3.** | Sorge vor negativen Folgen durch das Screening (z.B.: falsch-positive Befunde oder Überdiagnosen) |  |  |
| **4.** | Angst vor rechtlichen Folgen, wenn das Screening nicht durchgeführt wird, der Patient/die Patientin jedoch im Verlauf einen Lungenkrebs entwickelt. |  |  |
| **5.** | Es ist finanziell lukrativ. |  |  |
| **6.** | Aktuelle Empfehlungen in Leitlinien von akkreditierten medizinischen Gesellschaften. |  |  |
| **7.** | Die Erwartungshaltung des Patienten/ der Patientin  gegenüber Screening als effektive Methode im Kampf gegen Lungenkrebs. |  |  |

**Frage 3**

Wie hoch schätzen Sie den Anteil der Österreichischen Ärztinnen und Ärzte, die die Thorax-Computertomographie zum Lungenkrebsscreening bei Menschen die rauchen oder geraucht haben empfehlen?

Ca. _____%

**Frage 4**

Welche der folgenden Gründe beeinflussen die Entscheidung Ihrer Kolleginnen und Kollegen zur Empfehlung/ Nicht-Empfehlung einer Thorax-Computertomographie als Screening Methode für Lungenkrebs Ihrer Meinung nach?

|  |  | **Dies beeinflusst meine Entscheidung** | **Dies beeinflusst meine Entscheidung NICHT** |
| --- | --- | --- | --- |
| **1.** | Reduktion der Lungenkrebs Mortalität durch das Screening. |  |  |
| **2** | Reduktion der Lungenkrebs Inzidenz durch das Screening. |  |  |
| **3.** | Angst vor negativen Folgen durch das Screening (falsch positive Befunde, Überdiagnosen) |  |  |
| **4.** | Sorge vor rechtlichen Folgen, wenn das Screening nicht durchgeführt wird, der Patient/die Patientin jedoch im Verlauf einen Lungenkrebs entwickelt. |  |  |
| **5.** | Es ist finanziell lukrativ. |  |  |
| **6.** | Aktuelle Empfehlungen in Leitlinien von akkreditierten medizinischen Gesellschaften. |  |  |
| **7.** | Die Erwartungshaltung des Patienten/ der Patientin  gegenüber Screening als effektive Methode im Kampf gegen Lungenkrebs. |  |  |

**Screening-Wissen**

Stellen Sie sich Männer im Alter von 50 bis 74 Jahren vor, die aktuell rauchen oder geraucht haben und die über einen Zeitraum von ca. 10 Jahren entweder an der Früherkennung mittels Computertomographie (CT) oder nicht an dieser Früherkennung teilnehmen.

**QWF1a**

Wie viele von 1000 Männern, die KEINE regelmäßige Früherkennung mittels Computertomographie durchführen, werden Ihrer Meinung nach an Lungenkrebs in diesen 10 Jahren versterben?

_____ von 1000

**QWF1b**

Wie viele von 1000 Männern, die regelmäßige Früherkennung mittels Computertomographie durchführen, werden Ihrer Meinung nach an Lungenkrebs in diesen 10 Jahren versterben?

_____ von 1000

**QWF2a**

Denken Sie, dass Lungenkrebs Screening mittels Thorax Computertomographie dem Patienten auch schaden kann?

- Ja → QWF2b
- Nein → QWF3a

**QWF2b**

Welche Schäden könnte es geben?

____

**QWF3a**

Bei wievielen Männern ohne Lungenkrebs fand sich ein verdächtiger Herd, der ein weiteres CT, eine Gewbeentnahme (Biopsie) oder eine andere Diagnostik notwendig machte?

____von 1000

**QWF3b**

Bei wievielen Männern wurde ein nicht fortschreitender Tumor als fortschreitend diagnostiziert und unnötig behandelt?

____von 1000

**QWF4**

Denken Sie, dass der Nutzen des Lungenkrebs Screening mittels Thorax Computertomographie (Reduktion der Mortalität durch Lungenkrebs) den Schaden (Falsch positive Ergebnisse, Überdiagnosen) überwiegt?

- Ja
- Nein

**Intervention: Fact-Box**


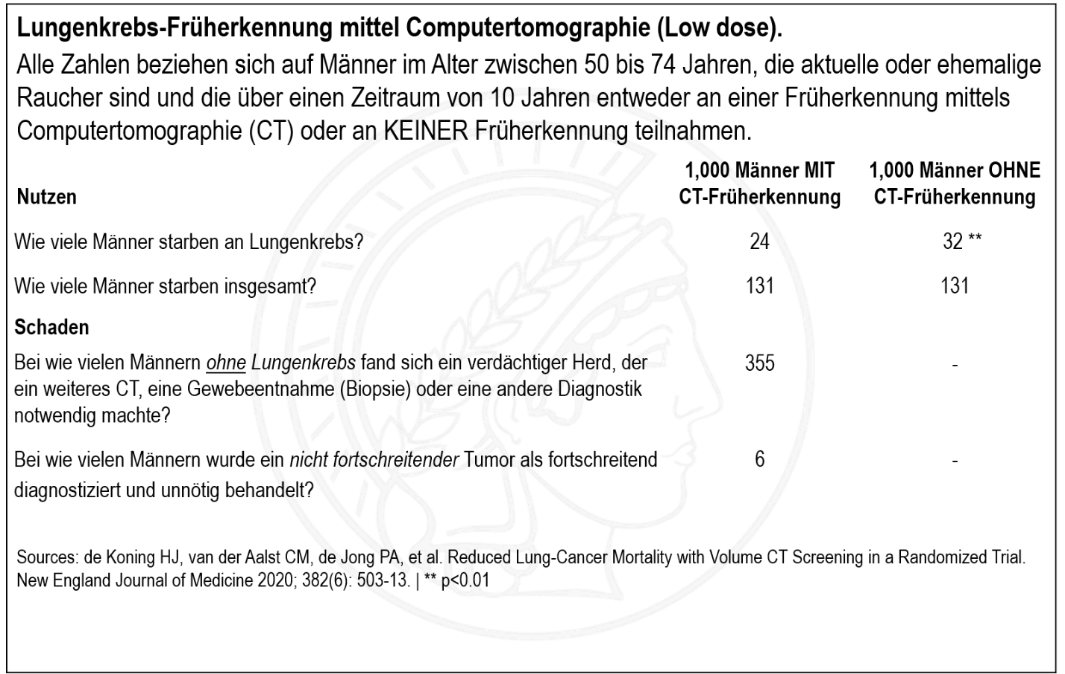


**Intervention: Icon Array**

**
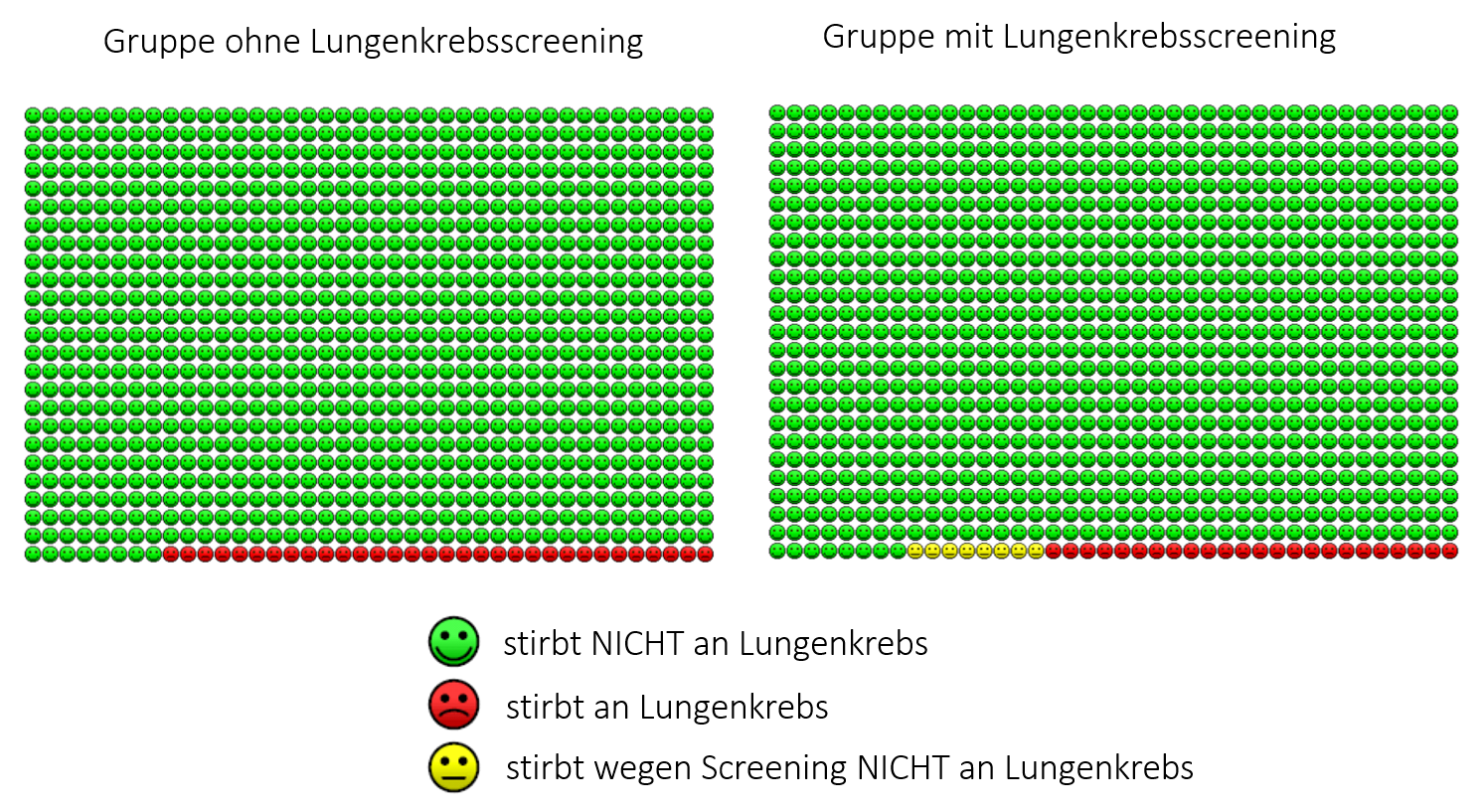
**

**QFB1a**

Hat sich Ihre Einstellung zu Nutzen und Schaden von Lungenkrebs Screening nach Lesen der Daten in der Fact-Box geändert?

- Ja → QFB1b
- Nein → Exit “Demography”

**QFB1b**

Wie viele von 1000 Männern, die KEINE regelmäßige Früherkennung mittels Computertomographie durchführen, werden Ihrer Meinung nach an Lungenkrebs in diesen 10 Jahren versterben?

_____ von 1000

**QFB1c**

Wie viele von 1000 Männern, die regelmäßige Früherkennung mittels Computertomographie durchführen, werden Ihrer Meinung nach an Lungenkrebs in diesen 10 Jahren versterben?

_____ von 1000

**QFB2a**

Denken Sie, dass Lungenkrebs Screening mittels Thorax Computertomographie dem Patienten auch schaden kann?

- Ja → QFB2b
- Nein → QFB3a

**QFB2b**

Welche Schäden könnte es geben?

____

**QFB3a**

Bei wie vielen Männern ohne Lungenkrebs fand sich ein verdächtiger Herd, der ein weiteres CT, eine Gewbeentnahme (Biopsie) oder eine andere Diagnostik notwendig machte?

____von 1000

**QFB3b**

Bei wie vielen Männern wurde ein nicht fortschreitender Tumor als fortschreitend diagnostiziert und unnötig behandelt?

____von 1000

**QFB4**

Denken Sie, dass der Nutzen des Lungenkrebs Screening mittels Thorax Computertomographie (Reduktion der Mortalität durch Lungenkrebs) den Schaden (Falsch positive Ergebnisse, Überdiagnosen) überwiegt?

- Ja
- Nein

**QGK1_4**

Welche der folgenden Aussagen beweist, dass Krebs Screening "Leben rettet"?

|  |  | **Beweist, dass Screening Leben rettet** | **Beweist nicht, dass Screening Leben rettet** |
| --- | --- | --- | --- |
| a. | Durch Screening diagnostizierter Krebs hat eine höhere 5-Jahres Heilungsrate als durch Symptome diagnostizierter Krebs. |  |  |
| b. | Die Diagnoseraten von Krebs sind bei gescreenten Personen höher als bei nicht gescreenten Personen. |  |  |
| c. | Durch Screening wird Krebs häufiger in frühen Stadien diagnostiziert als ohne Screening. |  |  |
| d. | Bei gescreenten Personen ist die Sterblichkeitsrate niedriger als bei nicht gescreenten Personen. |  |  |

**ENGLISH TRANSLATION OF SURVEY INSTRUMENT**

“Are the opinions of Austrian pulmonologists on lung cancer screening influenced by a Fact-Box?”

**Survey**

Dear Colleague,

Many medical decisions are not made in situations of complete certainty but fall into the numerous gray areas of probabilities. In this study, we want to investigate one of these areas and find out from you, as Austrian experts, how you evaluate lung cancer screening. Based on this survey, we aim to determine how the pros and cons of lung cancer screening should be best prepared and presented for all involved.

To answer this research question, we ask you to take a short time to answer questions about your attitude and recommendation practice regarding thoracic CTs as a screening method. Afterwards, you will receive a Fact-Box with numerical information about the benefits and harms of this method. Half of the participants will also receive the same information in addition to the Fact-Box, graphically processed in the form of a so-called Cates plot. After reviewing this information, we ask you to answer the questionnaire again. Your participation in this research project is voluntary. You can stop the survey at any time, without giving reasons.

All study participants will be coded with a consecutive number (pseudoanonymized). The data to be evaluated will be stored on a drive of the Karl Landsteiner Institute for Lung Research and Pneumological Oncology, Klinik Ottakring with restricted access, only coded with this number, and then evaluated. Only authorized persons have access to the original data. After the study is completed, the file will be deleted.

Thank you very much for participating in this survey!

**Recommendation Behavior/Reasons for Recommendation**

**Question 1**

Do you routinely recommend thoracic computed tomography as lung cancer screening for smokers and ex-smokers?

- Yes
- No

**Question 2**

Which of the following reasons influence your decision to recommend/ not recommend thoracic computed tomography as a screening method for lung cancer?

|  |  | **This influences my decision** | **This does NOT influence my decision** |
| --- | --- | --- | --- |
| **1.** | Reduction of lung cancer mortality through screening. |  |  |
| **2** | Reduction of lung cancer incidence through screening. |  |  |
| **3.** | Concern about negative consequences of screening (e.g., false-positive findings or overdiagnoses). |  |  |
| **4.** | Fear of legal consequences if screening is not performed, but the patient later develops lung cancer. |  |  |
| **5.** | It is financially lucrative. |  |  |
| **6.** | Current recommendations in guidelines from accredited medical societies. |  |  |
| **7.** | The patient's expectation of screening as an effective method in the fight against lung cancer. |  |  |

**Question 3**

What percentage of Austrian doctors do you estimate recommend thoracic computed tomography for lung cancer screening in people who smoke or have smoked?

Approximately _____ %

**Question 4**

Which of the following reasons do you think influence your colleagues' decision to recommend/ not recommend thoracic computed tomography as a screening method for lung cancer?

|  |  | **This influences my decision** | **This does NOT influence my decision** |
| --- | --- | --- | --- |
| **1.** | Reduction of lung cancer mortality through screening. |  |  |
| **2** | Reduction of lung cancer incidence through screening. |  |  |
| **3.** | Concern about negative consequences of screening (e.g., false-positive findings or overdiagnoses). |  |  |
| **4.** | Fear of legal consequences if screening is not performed, but the patient later develops lung cancer. |  |  |
| **5.** | It is financially lucrative. |  |  |
| **6.** | Current recommendations in guidelines from accredited medical societies. |  |  |
| **7.** | The patient's expectation of screening as an effective method in the fight against lung cancer. |  |  |

**Screening Knowledge**

Imagine 50 to 74-year old men, who are currently smoking or have smoked and who either participate in early detection using computed tomography (CT) or do not participate in this early detection over a period of about 10 years.

**QWF1a**

How many out of 1,000 men who do NOT undergo regular early detection using computed tomography in your opinion, will die of lung cancer in these 10 years?

_____ out of 1,000

**QWF1b**

How many out of 1,000 men who undergo regular early detection using computed tomography will, in your opinion, die of lung cancer in these 10 years?

_____ out of 1,000

**QWF2a**

Do you think lung cancer screening using thoracic computed tomography can also harm the patient?

- YES → QWF2b
- NO → QWF3a

**QWF2b**

What kind of harm could there be?

____

**QWF3a**

In how many men without lung cancer was a suspicious lesion found that required further CT, a tissue sample (biopsy), or other diagnostics?

____ out of 1,000

**QWF3b**

In how many men was a non-progressing tumor diagnosed as progressing and unnecessarily treated?

____ out of 1,000

**QWF4**

Do you think the benefit of lung cancer screening using thoracic computed tomography (reduction in lung cancer mortality) outweighs the harm (false positive results, overdiagnoses)?

- Yes
- No

**Intervention: Fact-Box**


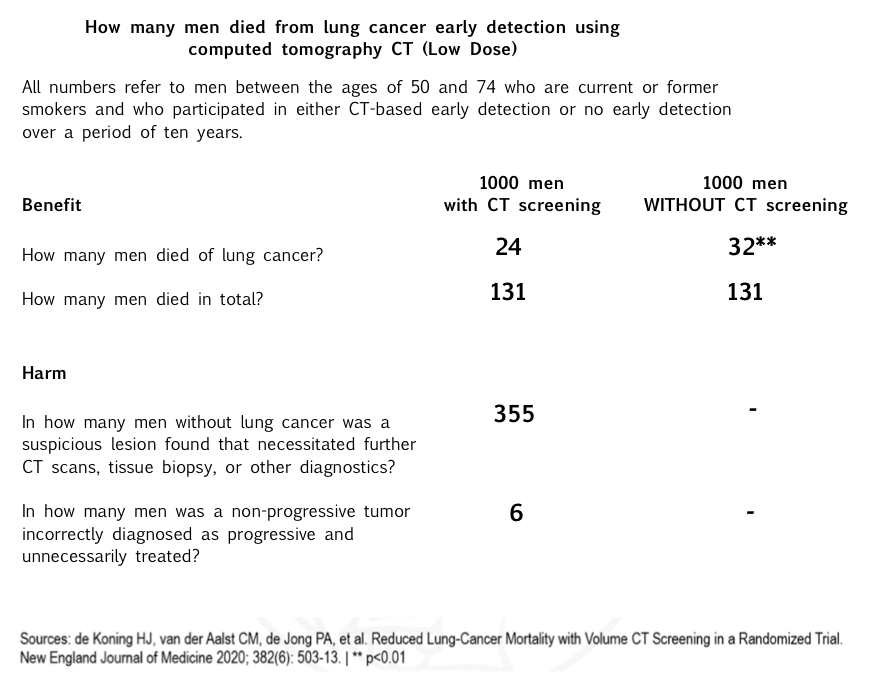


**Intervention: Icon Array**


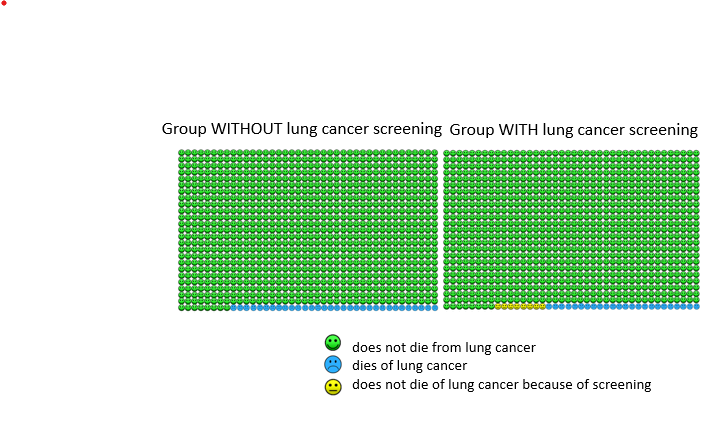


**QFB1a**

Has your opinion on the benefits and harms of lung cancer screening changed after reading the data in the Fact-Box?

- Yes → QFB1b
- No → Exit “Demography”

**QFB1b**

How many out of 1,000 men who do NOT undergo regular early detection using computed tomography will, in your opinion, die of lung cancer in these 10 years?

_____ out of 1,000

**QFB1c**

How many out of 1,000 men who undergo regular early detection using computed tomography will, in your opinion, die of lung cancer in these 10 years?

_____ out of 1,000

**QFB2a**

Do you think lung cancer screening using thoracic computed tomography can also harm the patient?

- Yes → QFB2b
- No → QFB3a

**QFB2b**

What kind of harm could there be?

____

**QFB3a**

In how many men without lung cancer was a suspicious lesion found that required further CT, a tissue sample (biopsy), or other diagnostics?

____ out of 1,000 QF3b

**QFB3b**

In how many men was a non-progressing tumor diagnosed as progressing and unnecessarily treated?

____ out of 1,000

**QFB4**

Do you think the benefit of lung cancer screening using thoracic computed tomography (reduction in lung cancer mortality) outweighs the harm (false positive results, overdiagnoses)?

- Yes
- No

**QGK1_4**

Which of the following statements proves that cancer screening "saves lives"?

|  |  | **Proves that screening saves lives** | **Does not prove that screening saves lives** |
| --- | --- | --- | --- |
| a. | Cancer diagnosed through screening has a higher 5-year cure rate than cancer diagnosed through symptoms. |  |  |
| b. | The diagnosis rates of cancer are higher in screened individuals than in non-screened individuals. |  |  |
| c. | Screening more frequently diagnoses cancer in early stages than without screening. |  |  |
| d. | The mortality rate is lower in screened individuals than in non-screened individuals. |  |  |
